# Clinical and epidemiological characteristics of multi-drug resistant *Enterobacterales* isolated from King Fahad Hospital of the University, AlKhobar, Saudi Arabia

**DOI:** 10.25122/jml-2023-0189

**Published:** 2024-01

**Authors:** Fadwa Bernawi, Aisha Alamri, Muzaheed, Amani Alnimr

**Affiliations:** 1 Microbiology Laboratory, King Faisal Specialist Hospital & Research Centre, Madinah, Kingdom of Saudi Arabia; 2 Department of Clinical Laboratory Sciences, College of Applied Medical Sciences, Imam Abdulrahman Bin Faisal University, Dammam, Kingdom of Saudi Arabia; 3 Department of Microbiology, College of Medicine, Imam Abdulrahman Bin Faisal University, Dammam, Kingdom of Saudi Arabia

**Keywords:** multi-drug resistant *Enterobacterales*, carbapenem-resistant strains, *Escherichia coli*, *Klebsiella pneumoniae*, Saudi Arabia

## Abstract

Multi-drug resistant (MDR) *Enterobacterales* remain a major clinical problem. Infections caused by carbapenem-resistant strains are particularly difficult to treat. This study aimed to assess the clinical and epidemiological characteristics of MDR *Enterobacterales* isolates. A total of 154 non-repetitive clinical isolates, including *Escherichia coli* (*n* = 66), *Klebsiella pneumoniae* (*n* = 70), and other *Enterobacterales* (*n* = 18), were collected from the Diagnostic Microbiology Laboratory at King Fahad Hospital of the University. Most *E. coli* isolates were collected from urine specimens (*n* = 50, 75.8%) and resistance against the third and fourth-generation cephalosporins (ceftriaxone, ceftazidime, cefixime, and cefepime) and fluoroquinolones (ciprofloxacin and levofloxacin) was assessed. Clonal relatedness analysis using enterobacterial repetitive intergenic consensus polymerase chain reaction (ERIC-PCR) revealed two clones (*E. coli* A and B), each comprising two strains. Most *K. pneumoniae* samples were collected from respiratory specimens (27.1%, 20 samples), and the strains showed overall resistance to most of the antimicrobials tested (54%‒100%). Moreover, clonal-relatedness analysis using ERIC-PCR revealed seven major clones of *K. pneumoniae*. These findings suggest nosocomial transmission among some identical strains and emphasize the importance of strict compliance with infection prevention and control policies and regulations. Environmental reservoirs could facilitate this indirect transmission, which needs to be investigated.

## INTRODUCTION

The Centers for Disease Control and Prevention (CDC) and the World Health Organization (WHO) have identified carbapenem-resistant *Enterobacterales* (CRE) as critical pathogens that require urgent global health attention [1,2]. Over the past decade, reports addressing carbapenem resistance have increased. Multiple risk factors are associated with the emergence and dissemination of carbapenem-resistant *Enterobacterales* [3]. Importantly, the frequent use of carbapenems to manage complicated and invasive infections caused by multidrug-resistant Gram-negative bacilli is considered the most important driver for the rise in CRE [4].

With their remarkable ability to resist antibiotics, MDR-*Enterobacterales* are often considered significant healthcare-associated pathogens. Their drug resistance is largely associated with intrinsic mechanisms or via acquiring a mobile genetic element that limits the utility of empirical therapy. Among members of *Enterobacterales*, carbapenem resistance is mainly linked to the expression of destructive enzymes such as *Klebsiella pneumoniae* carbapenemase (KPC) and oxacillin-hydrolyzing carbapenemase (OXA-48) that are often transferred through horizontal gene, making their control hard to achieve [5].

CRE are associated with hospital-acquired infections, leading to high economic costs, poor clinical outcomes, and a significant increase in the risk of mortality [6]. Effective antimicrobial agents and antibiotic susceptibility testing are the gold standards for their detection and treatment [7]. Identifying CRE carriers through epidemiological surveys and screening cultures is useful for identifying asymptomatic carriers [8]. Bacterial genotyping can help control the spread of pathogens by tracing the origin of outbreaks and is often referred to as molecular epidemiology [9].

Saudi Arabia faces the challenge of increasingly emerging CRE cases, with reports from local hospitals [10]. Therefore, the objective of this study was to assess the clinical and epidemiological characteristics of MDR *Enterobacterales* isolates from King Fahad Hospital of the University (KFHU) in the Eastern region of Saudi Arabia. The study also aimed to determine the genetic diversity of isolates using enterobacterial repetitive intergenic consensus polymerase chain reaction (ERIC-PCR).

## MATERIAL AND METHODS

### Bacterial isolation and identification

A total of 154 non-repetitive MDR Enterobacterales clinical isolates, including *E. coli* (*n* = 66), *K. pneumoniae* (*n* = 70), and other *Enterobacterales* (*n* = 18), were collected between January 2017 and March 2020 from the Diagnostic Microbiology Laboratory at King Fahad Hospital of the University. The samples were cultured on sheep blood and MacConkey agar and incubated at 37°C for 18‒24 h. Suspected colonies that tested positive for catalase and negative for oxidase were further confirmed through the VITEK (VITEK2 GN ID card) Mass Spectrometry (MS) system (Biomérieux, Craponne).

### Antimicrobial susceptibility testing

A battery of 15 antimicrobial agents was used to determine the antimicrobial susceptibility of the isolates using VITEK 2 and AST Card-GN99 (Biomérieux) as recommended by the Clinical Laboratory Standards Institute (CLSI 2019). The antimicrobials tested were ceftazidime (CAZ), meropenem (MRP), amoxicillin + clavulanate (AMC), cefixime (CXM), ceftriaxone (CTX), cefepime (FEP), imipenem (IMIP), ciprofloxacin (CIP), levofloxacin (LEVO), gentamicin (GENT), amikacin (AMIK), colistin (COLIS), tigecycline (TGC), and trimethoprim (TRIMETH). All strains collected in this study were MDR (non-susceptible to at least one agent in three or more antibiotic classes) [11]. Control strains, *E. coli* ATCC 25922, *Klebsiella pneumoniae* ATCC 700603, and *Pseudomonas aeruginosa* ATCC 27853, were included in every antibiotic susceptibility testing (AST) run [12]. All collected strains exhibited an MDR profile defined by the isolate being non-susceptible to at least one agent in ≥ three antimicrobial categories.

The minimum inhibitory concentration (MIC) was estimated for a subset of CRE strains using IMIP and MRP Etest strips (Biomérieux) following the instructions of the Clinical and Laboratory Standards Institute (CLSI) [13].

### ERIC-PCR

The genomic DNA of *E. coli* and *K. pneumoniae* was prepared by boiling the isolates and subsequently used as templates. Bacterial genomic DNA was extracted by emulsifying a loop full of bacteria from a Luria-Bertani agar plate (Fisher Scientific) into 300 µl of sterile, molecular biology-grade water. The tube was then incubated at 95°C in a heat block for 15 min and centrifuged at 13,000 rpm for 10 min. Two microlitres of the supernatant were used as a DNA template. ERIC-PCR was performed using a thermocycler (Bio-Rad) and ERIC primers; forward: 5'-ATG TAA GCT CCT GGG GAT TCAC-3' and reverse: 5'-AAG TAA GTG ACT GGG GTG AGC G3' [14]. The amplification was performed by adding a mixture (25 µL per reaction) of 12.5 µL GoTaq green master mix (M7122, Promega), 2 µL primers of each primer, 9.5 µL nuclease-free water, and 1 µL of DNA template. The PCR protocol consisted of an initial denaturation (94°C for 5 min) followed by 40 cycles of denaturation (95°C for 1 min), annealing (51°C for 1 min) followed by 65°C for 8 min, and a final extension (72 °C for 10 min) [14]. The PCR products were loaded onto a 1.5% SeaKem LE agarose gels (Lonza), with 1 KB and 100 Bp DNA ladder (MOLEQULE-ON) separated at a constant voltage of 90 V for 1.5 hours, and the banding patterns were visualized under ultraviolet radiation.

### Genotyping analysis

ERIC patterns were analyzed using GelClust software. The ERIC profiles were compared using the Dice method and clustered using the unweighted pair group method with the arithmetic mean program. Isolates with a similar pattern were considered one ERIC type. A dendrogram was then constructed based on these clusters.

### Data analysis

The clonal types of *K. pneumoniae* and *E. coli* were compared with the patient’s demographic and clinical data to identify possible epidemiological links (Table 1A). Similarly, comparisons were made with primary diagnoses and treatment strategies to identify clinical outcomes (Table 1B).

**Table 1A:** Characteristics of identical clones

Organism	Cluster	Identical clones	DATE ISOLATED	Age/Gen	Location	Specimen	I/C	Epidemiological link
***E. coli clones* - 2 pairs**	**A**	544	10-May-2019	72F	Outpatient	Urine	I	Not found
		553	15-May-2019	24F	Outpatient	Urine	I	Not found
	**B**	552	14-May-2019	58F	Outpatient	Urine	I	Not found
		642	19-Nov-2019	54M	Outpatient	Urine	I	Not found
**KP clones - 7 pairs**	**C**	493	30-Mar-2019	78F	Ward 1	Urine	C	Not found
		63	06-Feb-2017	48YM	ICU	Blood	I	Not found
	**D**	357	01-Dec-2018	26F	Ward 2	Skin	C	Possible link - case 337 had an interventional radiology procedure 'fluoroscopy guided NG tube insertion' on 19^th^ Nov
		337	15-Nov-2018	7y M	Ward 1	Rectal	C	Case 357 - another procedure in the same room on 28^th^ Nov
	**E**	631	07-Nov-2019	57F	ICU	Transtracheal	C	Same unit, separate beds/rooms
		645	21-Nov-2019	58M	ICU	Blood	I	No common procedures apart from intubation. Possibly environmental reservoir
	**F**	118	19-Apr-2017	3MO/F	ICU	Blood	I	Same unit, separate beds/rooms
		156	04-Aug-2017	78 y M	ICU	Blood	I	No common procedures apart from intubation. Possibly environmental reservoir
	**G**	571	25-May-2019	63F	ICU	Rectal	C	Same unit, separate beds/rooms
		495	31-Mar-2019	45F	ICU	Urine	C	No common procedures apart from intubation. Possibly environmental reservoir
	**H**	523	18-Apr-2019	76F	ICU	Blood	I	Not found
		662	01-Dec-2019	73M	ICU	Tracheal	C	Not found
		663	05-Dec-2019	92M	ICU	Tracheal	C	Not found
	**I**	32	10-Jan-2017	70M	ICU	Urine	I	Same unit, separate beds/rooms
		75	19-Feb-2017	22YM	ICU	Tracheal	I	No common procedures apart from intubation. Possibly environmental reservoir

**Table 1B:** Characteristics of identical clones

Organism	Cluster	Identical clones		Primary diagnosis/Co-morbidities		Treatment given		Outcome	
***E. coli* clones - 2 pairs**	**A**	544	T2DM, HT, Pulmonary HT, CKD IV, dyslipidemia, osteoporosis, hypothyroidism		Trimethoprim-sulpha		Recovered	
		553	Pregnant T1, no co-morbidities		Augmentin		Recovered	
	**B**	552	missing data		missing data		Missing data	
		642	Crohn disease		Ciprofloxacin		Deceased	
**KP clones - 7 pairs**	**C**	493	Recurrent UTI, T2DM, HT, CKD IV, hyponatremia		None		Recovered	
		63	Disseminated hydatid disease with ascites		Colistin, meropenem		Deceased	
	**D**	357	Post-CS surgical wound infection		Tazocin		Recovered	
		337	Post-meningitis hydrocephalus, VP shunt, cochlear sclerosis		None		Discharged	
	**E**	631	Stroke, peptic ulcer disease		None		Discharged	
		645	T2DM, HT, IHD, CHF, pleural effusion		Colistin, ceftazidime-avibactam		Recovered	
	**F**	118	Arnold Chiari malformation, hydrocephalus, VP shunt, biliary atresia, G6PD deficiency		Gentamicin, meropenem		Deceased	
		156	Septic shock, aspiration pneumonia, empyema, T2DM, HT, old stroke		Colistin, ceftazidime avibactam		Deceased	
	**G**	571	spondylodiscitis, previous tuberculosis, and brucellosis.		None		Discharged	
		495	Breast abscess, post-surgical catheter, repeated cultures negative		None		Discharged	
	**H**	523	Acute heart failure, Atrial fibrillation, T2DM, HT, dyslipidemia, acute renal failure, cervical atypia		None		Deceased	
		662	Stroke, dysphasia		None		Deceased	
		663	Influenza (H1N1), T2DM, HT, Interstitial lung disease, inguinal incarcinated hernia,		None		Deceased	
	**I**	32	Old stroke, sepsis		Colistin		Recovered	
		75	Trauma with multi-fractures		Colistin, ceftazidime-avibactam		Recovered	

Abbreviations: T2DM, Type 2 Diabetes Mellitus; HT, Hypertension; Pulmonary HT, Pulmonary Hypertension; CKD IV, Chronic Kidney Disease Stage IV; T1, Type 1 Diabetes Mellitus; UTI, Urinary Tract Infection; CS, Cesarean Section; VP Shunt, Ventriculoperitoneal Shunt; IHD, Ischemic Heart Disease; CHF,Congestive Heart Failure; G6PD Deficiency, Glucose-6-Phosphate Dehydrogenase Deficiency; ICU, Intensive Care Unit; OPD, Outpatient Department

## RESULTS

### Clinical isolates

The *E. coli* clinical isolates were isolated from clinical samples, including urine (50, 75.8%), blood (5, 7.6%), and other samples (11, 16.6%). *K. pneumoniae* strains were mostly collected from respiratory specimens, accounting for 19 samples (27.1%), followed by urine and blood specimens (*n* = 15, 21.4%; *n* = 11, 15.7%), respectively, and other samples (*n* = 17, 24%).

Among the 66 *E. coli* isolates identified, 43 were from women (65.2%) and 23 from men (34.8%). Additionally, the majority of the patients were aged 61 years and older, accounting for 42.4% of cases. This was followed by the age groups of 31–45 years (19.7%) and 46–60 years (18.2%). From the 70 *K. pneumoniae* strains isolated, most of the patients were men (*n* = 36, 51.4% vs. 48.6% women) and elderly subjects (*n* = 39, 55.7%).

### Antibiotic susceptibility patterns

Most strains were resistant to CAZ (55, 83.3%), AMC (42, 63.6%), CXM (64, 97%), CTX (61, 92.4%), FEP (47, 71.2%), CIP (55, 83.3%), LEVO (51, 77.3%), and TRIMETH (59, 89.4%). Similarly, 65 (98.5%), 50 (75.8 %), 61 (92.4%), 55 (83.3%), and 61 (92.4%) samples were susceptible to MRP, TAZ, IMIP, AMIK, and TGC, respectively. Thirty-three patients (50%) were equally susceptible or resistant to GEN antibiotics. Strains showed resistance to CAZ (68, 97.1%), MRP (22, 68.6%), AMC (65, 92.9%), TAZ (61, 87.1%), CXM (70, 100%), CTX (69, 98.6%), FEP (63, 90%), IMIP (46, 65.7%), CIP (63, 90%), LEVO (59, 84.3%), GEN (39, 55.7%), and TRIMETH (63, 90%). The isolates showed higher susceptibility to AMIK (38, 54.3%) and TGC (62, 88.6%) (Table 2).

**Table 2:** Antibiotic resistance pattern of MDR clinical isolates

Antibiotics	*E. coli* (*n* = 66)	*K. pneumoniae* (*n* = 70)
	Susceptible *n* (%)	Susceptible *n* (%)
CAZ	11 (16.7)	2 (2.9)
MRP	65 (98.5)	22 (31.4)
AMC	24 (36.4)	4 (5.7)
TAZ	50 (75.8)	9 (12.9)
CXM	02 (3)	0 (0)
CTX	04 (6.1)	1 (1.4)
FEP	19 (28.8)	5 (7.1)
IMIP	61 (92.4)	23 (32.9)
CIP	11 (16.7)	7 (10)
LEVO	15 (22.7)	11 (15.7)
GENT	33 (50)	31 (44.3)
AMIK	55 (83.3)	38 (54.3)
TGC	61 (92.4)	62 (88.6)
TRIMETH	07 (10.6)	6 (8.6)

* CAZ, Ceftazidime; MRP, Meropenem; AMC, Amoxicillin/Clavulanate; TAZ, Piperacillin/Tazobactam; CXM, cefotaxime; CTX, Ceftriaxone; FEP, Cefepime; IMIP, Imipenem; CIP, Ciprofloxacin; LEVO, Levofloxacin; GENT, Gentamicin; AMIK, Amikacin; TGC, Tigecycline; TRIMETH, Trimethoprim-sulfamethoxazole

** Two isolates were found intermediate resistant to FEP and one isolate was intermediate resistant to IMIP.

A subset of the CRE isolates collected after 2019 were further tested to determine the minimum inhibitory concentration of carbapenem. The MRP MICs against three *E. coli* strains (412, 537, and 547) were 0.75, 8, and > 32 µg/mL, respectively. These results were closely reflected in the IMIP MICs, which were 0.75, 2, and >32 µg/mL, respectively. In the case of 48 *K. pneumoniae* strains, the MRP MICs varied between 1.5 µg/mL to >32 µg/mL, while IMIP MICs ranged from 8 µg/mL to 32 µg/mL. Interestingly, MRP and IMIP showed comparable MIC readings, with 44 isolates (92%) showing carbapenem MICs > 32 µg/mL.

The number of non-survivors based on the 30-day mortality index was 13 (19.7%) compared to 53 survivors (80.3%) for *E. coli* isolates and 34 (48.6%) vs. 36 (51.4 %) for *K. pneumoniae* strains.

### Molecular fingerprinting through ERIC-PCR

**Figure 1:**
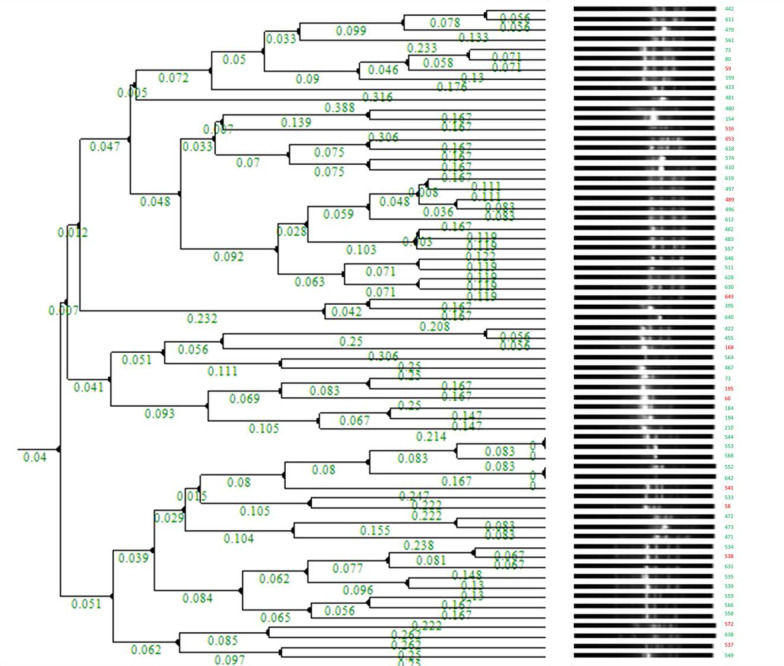
Cluster dendrogram of *E. coli* isolates * RED denotes YES for Mortality; GREEN denotes NO for Mortality

**Figure 2:**
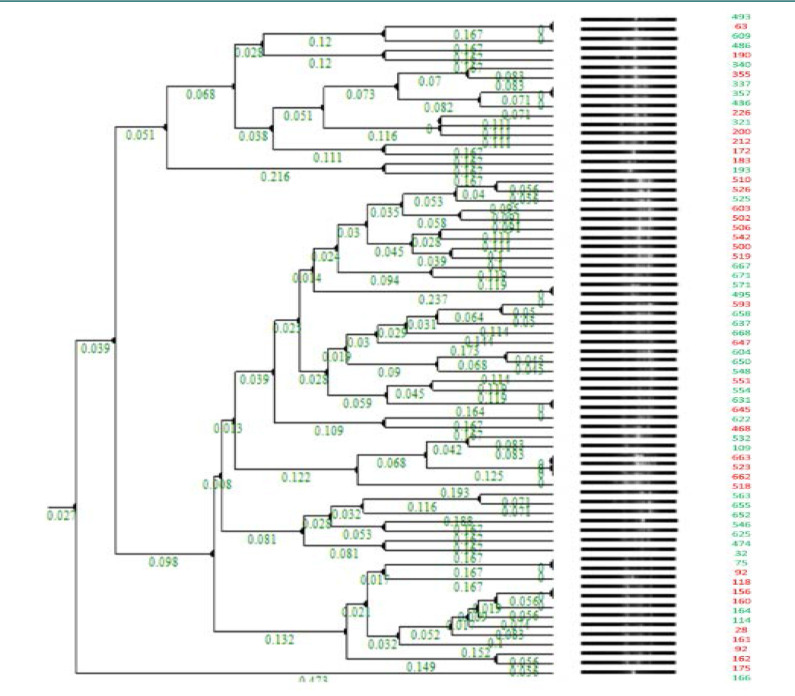
Cluster dendrogram of *K. pneumoniae* isolates * RED denotes YES for Mortality; GREEN denotes NO for Mortality

All *K. pneumoniae* and *E. coli* isolates were characterized through ERIC-PCR to determine their molecular fingerprints and phylogenetic relationships. Two pairs of *E. coli* clusters (544/553 and 552/642) were identified as identical clones (Figure 1), and multiple identical clones were found for *K. pneumoniae*, including 493/63, 571/495, 337/357, 663/523/662, 631/645, 32/75, 32/75, and 118/156 (Figure 2 and Table 1A).

## DISCUSSION

CRE have been associated with nosocomial infections and often result from a combination of factors, including weakened patient immunity, invasive medical procedures, improper hygiene practices, and antibiotic-resistant bacteria in healthcare settings. Consequently, this contributes to substantial economic costs, poor clinical outcomes, and a significant increase in the risk of mortality.

Urinary tract infections, surgical site infections, bloodstream infections, and respiratory infections are the most prevalent hospital-acquired infections. In this study, *E. coli* was most frequently isolated from the urine of young female participants, aligning with findings from a Mexican study that identified uropathogenic *E. coli* as a common cause of urinary tract infections, especially in women [15]. Additionally, *E. coli* strains demonstrated high resistance to advanced cephalosporins, which are among the most commonly prescribed antibiotics. However, most isolates retained good susceptibility to carbapenems (92‒98%). Fluoroquinolones are among the second-line antibiotics for acute uncomplicated cystitis, for which the strains included in the study exhibited high resistance (CIP, 83.3%; LEVO, 77.3%). Similar results were published by Kourtis *et al*., who reported reduced susceptibility to third-generation cephalosporins (17%) and fluoroquinolones (35%) and less than 1% resistance to carbapenems [16]. A study conducted in western Saudi Arabia revealed similar findings, where 95% of the isolates were resistant to extended-spectrum cephalosporins, including CTX, CAZ, and FEP, with high fluoroquinolone resistance [17].

The majority of *K. pneumoniae* samples were obtained from elderly male patients, predominantly from respiratory and urine specimens, with 19 and 17 cases, respectively. In our cohort, most isolates had high levels of resistance to most of the antimicrobials tested, including carbapenem with IMIP (65.7%). A multicentre study in Spain revealed high resistance to carbapenems (28‒96.9%) and other classes of antibiotics against MDR *K. pneumoniae*, with more samples isolated from the blood than from urine [18]. Similarly, research carried out in the Asser region of southern Saudi Arabia observed comparable patterns of resistance among 276 tested patients, where most samples were collected from the respiratory tract (61%). The isolates were highly resistant to IMIP and MRP (55.5% and 61.7%, respectively), in addition to extended-spectrum agents (CAZ, 92.5%) [19].

MIC estimation is a valuable tool for clinicians to manage complicated infections caused by multidrug-resistant organisms. In this study, we estimated the MIC for carbapenems (MRP and IMIP) in a subset of isolates that demonstrated qualitative carbapenem resistance. A comparative analysis with global studies highlights the variability in carbapenem resistance. For instance, a study from China on *K. pneumoniae* across 105 strains found that around 30% of these isolates produced carbapenemase (KPC), with 42% showing a MIC for IMIP of ≥16 µg/mL [20]. This contrasts with findings from Brazil, where an MDR-KP strain exhibited MRP and IMIP MICs of ≤ 4 µg/mL, except for two strains that showed significantly higher MICs (128 and 256 µg/mL, respectively), indicating the presence of bla_KPC_ among CRE isolates [21].

Carbapenem MICs have been evaluated in various regions of Saudi Arabia. For instance, In Riyadh city, the antibiotic resistance profile of MDR-KP and the carbapenem MICs for most strains were 16 µg/mL [22]. Similarly, a recent report from Jeddah, Saudi Arabia, showed elevated MRP and IMIP MICs (above 16 g/mL) in CRE *K. pneumonia* isolates co-harbouring OXA-48 and New Delhi metallo-beta lactamase (NDM) carbapenemases [23]. However, the studies mentioned above targeted MDR-KP isolates (similar to our study), and not all *K. pneumonia* strains were received in the microbiological laboratory. In our study, *K. pneumoniae* strains showed similar MIC readings, with 44 isolates (92%) showing carbapenem MICs (MRP and IMIP) > 32 µg/mL.

In a recent nationwide multicentre study conducted over two years, patients who tested positive for CRE were evaluated. The study included 189 patients, predominantly male participants, with an age range exceeding 62 years. Complicated UTIs and pneumonia accounted for most infections (23.8%). *K. pneumoniae* isolates accounted for most cases, followed by *E. coli* (87.3% vs. 11.1%), with both pathogens present in two individuals. The incidence of CRE bacteremia was 40.7%. The 30-day mortality rate for all the cases under investigation was 30.4%. This study further identified pneumonia and bacteremia as predictors of the 30-day mortality index [24]. In another study from Asia, the blood isolates of carbapenem-resistant *K. pneumoniae* were retrospectively evaluated. The 30-day mortality rate was 52.1% (89 samples) [25]. However, that study focused on blood isolates, whereas our study included all spectra of specimens that fulfilled the criteria for MDR, and the 30-day mortality rate among patients with MDR-KP in our study was 48.6%.

Genotyping results for MDR *E. coli* revealed major clusters with two identical clones: 544/553 and 552/642 (Figure 1). Urine samples 544 and 553 were collected in May 2019; however, sample 544 was collected from an elderly female who visited the emergency department, and sample 553 was from a young female who visited the outpatient department (OPD) five days later (Table 1A). The antibiotic resistance pattern differed slightly from sample 544, which was more resistant to the antibiotics tested (Table 3). In contrast, samples 552 and 642 were urine samples but collected within a 6-month timeframe (May-November 2019) from an outpatient clinic at KFHU. The short time span between the two samples suggests clonal spread, possibly because of shared surfaces or medical devices in the outpatient space. Additionally, the difference in the AST profiles may be attributed to the fitness cost of the resistant element (plasmid or conjugative transposons), as resistant determinants may be lost under selective pressure. Using ERIC-PCR as a discriminatory tool, a study from Ghana revealed a clonal spread from samples (urine and wound) for NDM-positive *E. coli* isolates and 2 OXA-48 *K. pneumonia* [26]. In another study from India targeting MDR *E. coli*, a range of major clusters was identified with multiple identical clones [27]. A local study in the western region of Saudi Arabia investigated the clonal relatedness among 211 extended-spectrum β-lactamase-producing *E. coli* cohorts using ERIC-PCR and a more advanced genotyping tool, multilocus sequence typing (MLST) where 32 sequence types (STs) were identified (ST131, ST38, and a novel ST8162 clone) [28].

**Table 3:** Characteristics of identical clones

Organism	Cluster	Identical clones	CAZ	MRP	AMC	TAZ	CXM	CTX	FEP	IMP	CIP	LEVO	GENT	AMIK	TGC	TRIMETH	MIC-Mero
***E. coli* clones - 2 pairs**	**A**	544	R	S	R	R	R	R	S	S	R	R	S	S	S	S	-
		553	S	S	S	S	R	R	S	S	S	S	R	S	S	R	-
	**B**	552	R	S	R	S	R	R	R	S	S	S	S	S	S	R	-
		642	S	S	S	S	R	R	S	S	S	S	S	S	S	R	-
**KP clones - 7 pairs**	**C**	493	R	R	R	R	R	R	R	R	R	R	R	R	S	R	MIC > 32
		63	R	R	R	R	R	R	R	R	R	R	S	S	S	R	MIC > 32
	**D**	357	R	S	R	S	R	R	R	S	R	S	R	S	S	R	-
		337	R	R	R	R	R	R	R	R	R	R	R	R	S	R	MIC > 32
	**E**	631	R	R	R	R	R	R	R	R	R	R	R	R	S	R	MIC > 32
		645	R	R	R	R	R	R	R	R	R	R	R	R	S	R	MIC > 32
	**F**	118	R	R	R	R	R	R	R	R	R	R	S	S	S	R	MIC > 32
		156	R	R	R	R	R	R	R	R	R	R	S	S	S	R	MIC > 32
	**G**	571	R	S	R	R	R	R	R	S	R	S	R	S	S	R	-
		495	R	S	R	S	R	R	R	S	S	S	R	S	S	R	-
	**H**	523	R	R	R	R	R	R	R	R	R	R	S	S	S	S	MIC > 32
		662	R	R	R	R	R	R	R	R	R	R	R	R	S	R	MIC > 32
		663	R	R	R	R	R	R	R	R	R	R	S	S	R	R	MIC > 32
	**I**	32	R	R	R	R	R	R	R	R	R	R	S	S	S	R	MIC > 32
		75	R	R	R	R	R	R	R	R	R	R	R	R	S	R	MIC > 32

Interestingly, the clustering of MDR-KP showed multiple clones within a close timeframe (Figure 2), such as 357 and 337 (invasive strains), 631 and 645 (inpatients carrying invasive strains), 662 and 663 (respiratory isolates), and 32 and 75 (urine and respiratory samples, respectively) (Table 1A). In cluster D (357 and 337), both patients underwent interventional radiology procedures (fluoroscopy-guided nasogastric tube insertion), whereas the other *K. pneumonia* clusters were mostly linked to the presence of patients in the intensive care unit (ICU) setting with potential environmental transfer of the same MDR strain. A range of comorbidities were noted in patients with identical clones (Table 1B), such as diabetes, hypertension, and pulmonary and cardiac conditions [29]. This finding is supported by studies that reported multiple healthcare-related outbreaks, particularly in high-risk settings such as ICUs [30]. Alternatively, in a major study from China, Kundu *et al*. analyzed 137 *K. pneumoniae* clinical isolates over two years (2019 and 2020). Their study revealed that neither major outbreaks nor clustering of strains was noted in the strains collected from various hospital wards [31]. Additionally, a study from Egypt found no major clusters in a CRE *K. pneumoniae* cohort. The authors justified this finding based on the heterogeneity among *K. pneumoniae* serotypes (variation in nucleotide sequences within the species) [32].

Although patient-to-patient cross-transmission is often linked to hospital outbreaks, environmental reservoirs, such as sinks and waste material, have been frequently implicated in the chain of infection [33]. Our study highlights the importance of using the 30-day mortality index to evaluate patient outcomes. The use of other parameters, such as case fatality risk, could provide insights into the different attributes of mortality. Furthermore, the association between antibiotic resistance and mortality rate must be further explored. Multivariate analysis using random variables should be performed to accurately assess the independent predictors of treatment failure or death. Additionally, genotypic characteristics (resistance genes, such as KPC and other carbapenem-resistant genes) that could explain the AST profile are lacking. Environmental sampling of closely related clusters must be assessed to confirm the potential clonal spread.

## CONCLUSION

Our current study analyzed the microbiological and clinical features of important MDR *Enterobacterales*, focusing on the most frequently isolated species, *E. coli* and *K. pneumoniae*. Our study reported a low CRE rate. However, different colonies may have different antibiotic resistance patterns, leading to difficulties in treating infections. We recommend future studies to evaluate genetic diversity in large geographical areas with a large number of clinical samples from the *Enterobacterales* family. Multivariate analysis using a random variable should be performed to accurately assess the independent predictors of treatment failure or death and find possible relationships between ERIC type and variables. The spread of MDR strains is a major global concern, and national surveillance programs conducted at regular intervals are essential to track these hard-to-treat strains.

## Data Availability

The datasets analyzed during the current study are available from the corresponding author upon reasonable request.

## Personal thanks

The authors would like to thank Prof. Niaz Ahmad, University of Hyderabad (UoH), India, for assisting in analyzing gels and deducing dendrograms.
